# Eccrine Squamous Syringometaplasia Mimicking Acute Cutaneous GVHD in a Pediatric HSCT Recipient: Case Report and Brief Review of the Indexed Literature

**DOI:** 10.1111/cup.70112

**Published:** 2026-04-20

**Authors:** Benedetta Galli, Cecilia Catapano, Iacopo Ghini, Mariateresa Rossi, Marina Venturini, Valeria Boccaletti

**Affiliations:** ^1^ Department of Dermatology and Venereology ASST Spedali Civili di Brescia, University of Brescia Brescia Italy; ^2^ Department of Pathology ASST Spedali Civili di Brescia, University of Brescia Brescia Italy

**Keywords:** eccrine ducts, eccrine squamous syringometaplasia, GVHD mimic, hand–foot syndrome, HSCT, pediatric dermopathology, syringometaplasia, toxic erythema of chemotherapy

## Abstract

Eccrine squamous syringometaplasia (ESS) is an uncommon reactive alteration of eccrine ducts, most often reported in oncologic and transplant settings, where it may clinically mimic acute cutaneous graft‐versus‐host disease (GVHD). We describe a 3‐year‐old boy with chronic granulomatous disease who developed a diffuse erythematous eruption 6 weeks after haploidentical hematopoietic stem cell transplantation. Clinically suggestive of acute GVHD, histopathologic examination instead revealed spongiotic dermatitis with prominent squamous metaplasia of eccrine ducts, in the absence of interface dermatitis, keratinocyte apoptosis, or satellitosis, supporting a diagnosis of ESS. The eruption resolved with topical corticosteroids without modification of systemic therapy. We also provide a brief review of Scopus/PubMed‐indexed literature, summarizing reported clinical settings, triggers, histopathologic features, and outcomes. Clinicopathologic correlation is essential in transplant recipients to distinguish ESS from GVHD and to avoid unnecessary escalation of immunosuppression.

## 1 Case Report

### 1.1 Case Description

A 3‐year‐old boy with chronic granulomatous disease caused by a splice‐site mutation in the *CYBB* gene (c.483+1G>A), cytochrome b‐245 beta chain, which encodes a critical subunit of the phagocyte NADPH oxidase complex required for reactive oxygen species generation and microbial killing, underwent a second allogeneic hematopoietic stem cell transplantation (HSCT) from his haploidentical father following progressive decline of donor chimerism after a previous transplant. Conditioning included alemtuzumab, busulfan, fludarabine, and single‐fraction total body irradiation, with posttransplant cyclophosphamide for GVHD prophylaxis.

The early posttransplant course was complicated by viral reactivations (rhinovirus/enterovirus DNA positive until 4 weeks after, CMV DNA positivity until 6 weeks after) and a transplant‐associated thrombotic microangiopathy with systemic capillary leak, which required treatment with corticosteroids, mycophenolate mofetil, ruxolitinib, and complement inhibition with eculizumab. In parallel, the patient received prolonged antiviral and antifungal therapies, including ganciclovir and voriconazole.

**FIGURE 1:**
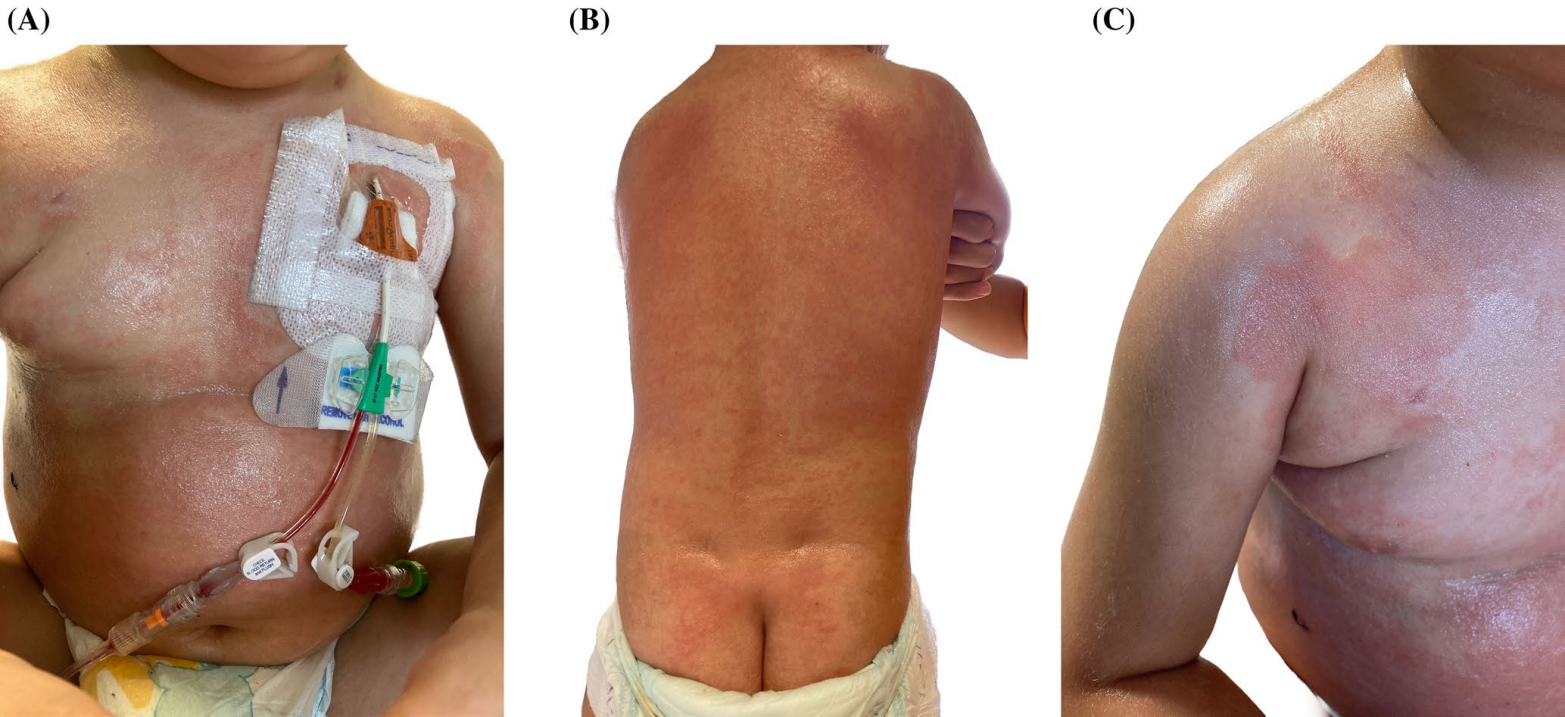
Clinical presentation of eccrine squamous syringometaplasia. Diffuse eczematous‐like eruption with confluent erythematous patches involving the abdomen and back (A, B), associated with mild infiltration and minimal follicular accentuation, without surface scaling. A sharply demarcated erythematous plaque is noted on the right anterior shoulder (C).

Approximately 6 weeks after HSCT, he developed a new cutaneous eruption. Clinically, this was characterized by multiple erythematous and infiltrated plaques, sharply demarcated and in part confluent, localized mainly to the abdomen and extending over the trunk. The surface was smooth to slightly scaly, without vesiculation, erosion, or atrophy. The abrupt onset and distribution were highly suggestive of acute cutaneous graft‐versus‐host disease (GVHD), and skin biopsy was therefore performed (Figure 1A–C).

**FIGURE 2:**
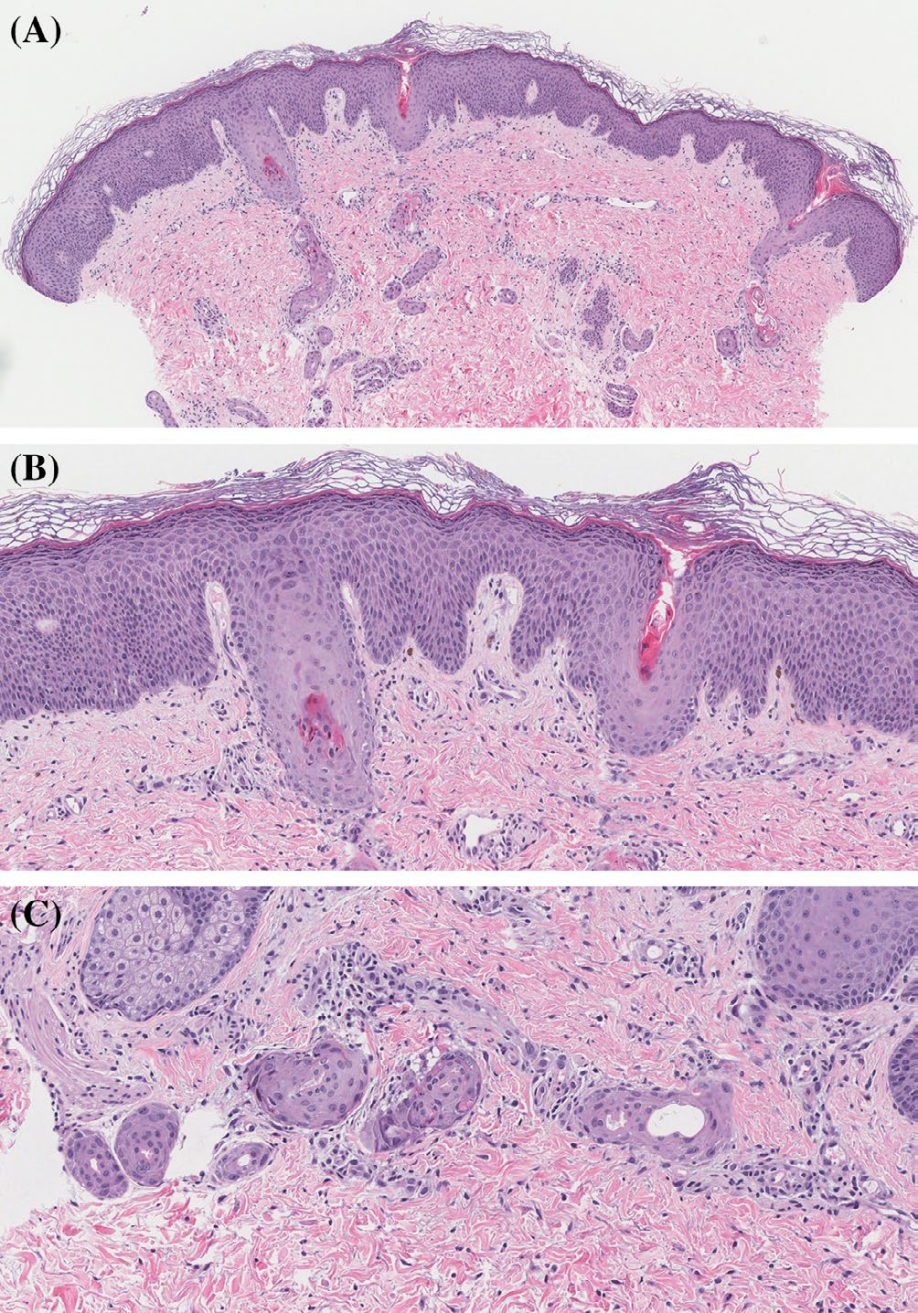
Histopathologic features of eccrine squamous syringometaplasia. Spongiotic and mildly acanthotic epidermis with prominent squamous metaplasia of eccrine ducts, particularly involving the ductal portion and extending to the acrosyringeal ostia (H&E, 2×) (A). At higher magnification, eccrine ducts show multilayered squamous epithelium with keratinization, while changes become less pronounced in the deeper secretory coils (H&E, 10×) (B). A mild mononuclear inflammatory infiltrate with predominantly perivascular and focal interstitial distribution is present in the superficial dermis (H&E, 20×) (C).

Histopathological examination of the abdominal specimen revealed spongiotic dermatitis associated with eccrine squamous syringometaplasia (ESS). The epidermis showed intercellular edema and focal parakeratosis. The most distinctive finding was the squamous metaplasia of eccrine ducts, where the normal cuboidal epithelium was replaced by multilayered squamous epithelium with keratinizing features. The changes were circumscribed to the eccrine apparatus and were accompanied by a mild, perieccrine lymphohistiocytic infiltrate. There were no apoptotic keratinocytes, no basal vacuolar alteration, and no satellitosis, and adnexal structures were preserved. Dermal collagen bundles were unremarkable, and there was no lichenoid interface reaction. The absence of hallmarks of GVHD allowed a confident clinicopathologic diagnosis of a toxic erythema of chemotherapy‐like eruption, with ESS as the predominant adnexal reaction pattern, a reactive and non‐alloimmune process (Figure 2A–C).

No changes were made to the ongoing systemic regimen; a topical corticosteroid was introduced, leading to progressive improvement. The eruption resolved gradually over subsequent weeks, with complete restitution of the skin and no pigmentary or cicatricial sequelae. No recurrence was documented during follow‐up.

This case underscores the diagnostic complexity of posttransplant eruptions in pediatric recipients. The clinical overlap between acute GVHD and other reactive dermatoses is considerable, as GVHD may closely mimic a range of cutaneous conditions. In such settings, reliance on clinical features alone may be insufficient, making management particularly challenging. In the present case, histological confirmation allowed for a conservative approach and was followed by a favorable dermatological outcome. More broadly, this observation highlights the importance of systematic histological assessment of GVHD‐suspect eruptions and of incorporating dermatopathological expertise into transplant care, to support balanced clinical decision‐making in an inherently complex context.

## 2 Review of the Literature

### 2.1 Rationale and Objectives

ESS is a duct‐centric eccrine reaction pattern within the spectrum of toxic erythema of chemotherapy that may closely simulate acute cutaneous GVHD in HSCT recipients. The earliest description by King and Barr in 1979 highlighted mucinous and squamous syringometaplasia involving eccrine ducts, while later reports by Hurt and by Metcalf and Maize placed ESS within chemotherapy‐associated eccrine injury and inflammatory panniculitides [1, 2, 3, 4]. Syringometaplasia is now regarded as a stereotyped adnexal response to cytotoxic, ischemic, or inflammatory stress rather than a standalone disease [5]. In this article, we use ESS for predominantly ductal squamous metaplasia and SMSE when metaplasia extends into the secretory coil; in our case, the change is duct‐predominant with subtle secretory involvement, that is, an ESS/SMSE pattern. Our objectives were to summarize the clinicopathologic spectrum of ESS, emphasize its distinction from acute GVHD and related adnexotropic toxicities, and provide a consolidated, indexed bibliography with attention to pediatric cases.

### 2.2 Methods

We searched Scopus and MEDLINE/PubMed from inception to August 30, 2025, using predefined terms. We included human cutaneous ESS with histopathologic confirmation and extracted trigger/exposure, distribution, histology, management/outcome, and age group; non‐cutaneous, non‐indexed, non‐eccrine, animal‐only, and image‐only entries were excluded. Eligible cases are summarized in Table 1, including additional indexed reports not individually cited in the narrative text [6, 7, 8, 9, 10].

**TABLE 1:** PubMed‐indexed reports including eccrine squamous syringometaplasia (ESS) with clinicopathologic data identified by our literature search and meeting the predefined inclusion criteria (human cutaneous ESS with histopathologic confirmation, as detailed in Section 2.2).

#	First author (year)	Journal	Number of patients (*n*)	Age group	Underlying setting	Trigger/specific drug(s)	Distribution/pattern	Timing of eruption (days after trigger)	Key histology note	PMID/identifier
1	King (1979)	*Journal of Cutaneous Pathology*	NR	Mainly adults	Mixed inflammatory/traumatic dermatoses	Local chronic injury/inflammation	Localized to involved plaques	NR	Mucinous and squamous syringometaplasia variants in eccrine ducts	500875
2	Hurt (1990)	*Archives of Dermatology*	NR (case series)	Adult (NR)	Oncologic chemotherapy	Various cytotoxic regimens	Intertriginous and acral, TEC‐like	NR	Duct‐centric squamous metaplasia within eccrine units	2404465
3	Metcalf (1990)	*American Journal of Dermatopathology*	NR	Adult (NR)	Lobular panniculitis/pyoderma gangrenosum	Inflammatory panniculitis	Ulcer edges, inflamed plaques	NR	Squamous syringometaplasia adjacent to panniculitis/PG	2331048
4	Serrano (1993)	*Journal of Cutaneous Pathology*	NR	Adults (NR)	Inflammatory dermatoses and papillomatous lesions	Local inflammatory conditions	Localized to lesions	NR	Eccrine duct squamous metaplasia in chronic inflammatory lesions	7682228
5	Abbas (2016)	*International Journal of Dermatology*	Review	—	Narrative review	Multiple triggers summarized	—	—	Review of syringometaplasia variants and mechanisms	26460811
6	Valks (1996)	*British Journal of Dermatology*	NR	Adult (NR)	Chemotherapy	Cytotoxic chemotherapy	Intertriginous areas	NR	Prominent squamous metaplasia of eccrine ducts	8736357
7	Valks (1997)	*Archives of Dermatology*	10	Adults	HSCT conditioning	Myeloablative regimens	Bilateral intertriginous and acral erythema	NR	ESS within chemotherapy‐associated eccrine injury	9236526
8	Martorell‐Calatayud (2011)	*Journal of the American Academy of Dermatology*	21	Mixed	Chemotherapy‐related bilateral dermatitis	Pegylated liposomal doxorubicin	Symmetric flexural/intertriginous plaques	2–30 days	Eccrine duct squamous metaplasia with apoptosis	21489654
9	Wong (1993)	*Archives of Dermatology*	1	Adult	Chemotherapy for melanoma	Cytotoxic chemotherapy	Generalized papulovesicular eruption	NR	ESS component within toxic changes	8436012
10	Sommer (1998)	*Journal of Cutaneous Pathology*	3	Children	Pediatric burns	Thermal injury	Burned skin areas	~10 days	ESS in burned skin with ductal squamous change	9508345
11	Thomas (2008)	*Pediatric Dermatology*	1	Pediatric	Rhabdomyosarcoma	Vincristine	Acral/intertriginous	NR	ESS without neutrophilic coil injury	19067868
12	Bittar (2018)	*Pediatric Dermatology*	1	Infant	HSCT conditioning	Busulfan, fludarabine, ATG	Axillae, inguinal folds	~28 days	Dyskeratotic cells and focal ESS	2923 256
13	Sanborn and Sauer (2008)	*Dermatologic Clinics*	Review	—	Chemotherapy reactions	Multiple agents	Various	1–3 weeks typical	Review of chemotherapy‐induced skin toxicities	—
14	Story (2012)	*Journal of the American Academy of Dermatology*	1	Adult	Targeted melanoma therapy	Vemurafenib	Intertriginous/TEC‐like	NR	Duct‐centric squamous metaplasia	23062917
15	Lescoat (2013)	*Dermatology*	1	Adult	Metastatic melanoma therapy	Vemurafenib	Flexural eruption	NR	ESS centered on eccrine ducts	23860306
16	Liuti (2013)	*Journal of the American Academy of Dermatology*	1	Adult	Melanoma therapy	Dabrafenib	Flexural rash	NR	Eccrine duct squamous metaplasia	23597777
17	Moioli (2016)	*Journal of Cutaneous Pathology*	1	Adult	Vemurafenib + radiotherapy	Vemurafenib	Radiation field	NR	Ductal squamous metaplasia in recall eruption	27094584
18	Garcia‐Navarro (2008)	*Archives of Dermatology*	1	Adult	Oncologic chemotherapy	Pegylated liposomal doxorubicin	Intertriginous areas	NR	Ductal squamous metaplasia in flexures	18936417
19	Kim (2015)	*Acta Dermato‐Venereologica*	1	Adult	Solid tumor chemotherapy	TS‐1	Localized to syringoma	NR	ESS within syringoma	25766447
20	Santosa (2017)	*JAAD Case Reports*	1	Adult	NSCLC chemotherapy	Pemetrexed	Localized pseudocellulitis‐like plaque	NR	Focal ductal squamous metaplasia	28229122
21	Huh (2017)	*Annals of Dermatology*	1	Adult	NSAID exposure	Pelubiprofen	Intertriginous eruption	NR	Eccrine duct squamous metaplasia	28566912
22	Molina‐Ruiz (2012)	*International Journal of Dermatology*	1	Adult	Psychiatric therapy	Olanzapine	Trunk/flexures	35 days	Squamous metaplasia of eccrine ducts	22257902
23	Korver (2006)	*Journal of Drugs in Dermatology*	1	Adult	Oncologic chemotherapy	Pegylated liposomal doxorubicin	Intertrigo‐like eruption	NR	ESS in flexural skin	17039658
24	Gallo (2013)	*Journal of Cutaneous Pathology*	3	Adults	Chemotherapy extravasation	Docetaxel	Infusion site	NR	Localized ESS at extravasation site	23170995
25	Baysse (2003)	*La Revue de Médecine Interne*	1	Young adult	Renal transplant with CMV	CMV infection	Trunk and limbs	NR	ESS with evidence of CMV	12814829
26	Alonso (2006)	*International Journal of Dermatology*	1	Adult	Suspected herpetic infection	Viral/drug‐related	Localized eruption	NR	Ductal squamous metaplasia mimicking HSV	16796646
27	Helton (1995)	*American Journal of Dermatopathology*	1	Adult	Annular elastolytic granuloma	Chronic inflammatory dermatosis	Photoexposed plaques	Chronic	Focal squamous syringometaplasia	8600809
28	Kwakman (2020)	*Cancers*	Review	—	Hand–foot syndrome	Various cytotoxics	Acral ± flexural	Days–weeks	Review on management of HFS/TEC	32053904
29	Reynaert (1992)	*Bone Marrow Transplantation*	1	Adult	Allogeneic BMT	Cytotoxic chemotherapy	Acral erythema	NR	TEC features; ESS discussed	1388082
30	Yeh (2011)	*Journal of Cutaneous Pathology*	10	Mostly adults	Chemotherapy‐associated injury	Various regimens	TEC‐like	1–3 weeks typical	Eccrine hidradenitis sine neutrophils; ductal changes	20506044
31	Tan (2013)	*Journal of Skin Cancer*	Review	—	SCC simulators	Various	—	—	ESS cited as SCC pitfall	23878739
32	Rongioletti (1991)	*Journal of Cutaneous Pathology*	1	Adult	Chemotherapy	Cytarabine	Acral erythema	NR	Necrotizing ESS	1774355
33	Rongioletti (1992)	*Journal of the American Academy of Dermatology*	1	Adult	Chemotherapy‐induced acral erythema	Cytotoxic chemotherapy	Acral	NR	Focal squamous metaplasia	1552086
34	Bordel (2005)	*Actas Dermo‐Sifiliográficas*	1	Adult	Chemotherapy extravasation	Cytotoxic chemotherapy	Infusion site	NR	Localized ESS	16476276
35	Braunstein (2014)	*Journal of Cutaneous Pathology*	1	Adult	Vemurafenib + radiation	Vemurafenib	Radiation recall	NR	Interface dermatitis with ductal squamous metaplasia	24517243
36	Yu (2015)	*American Journal of Dermatopathology*	1	Adult	Vemurafenib therapy	Vemurafenib	Flexural/TEC‐like	NR	Syringometaplasia with adnexal duct transformation	25839889

*Note:* Entries are listed in chronological order and summarize, for each report, the main clinical context/trigger, age group, distribution pattern, key histologic features, and bibliographic identifier. Abbas (2016) and Sanborn (2008) are narrative reviews included for mechanistic and timing context rather than for primary case material.

## 3 Results

### 3.1 Contexts and Triggers

Across the literature, ESS is most often reported in association with high‐dose chemotherapy and HSCT conditioning. Cytarabine‐based regimens, anthracyclines, and other cytotoxics can induce TEC‐like eruptions in which ESS represents the dominant adnexal reaction pattern, and several series document intertriginous or acral involvement in transplant recipients where ESS clinically overlaps with acute GVHD [2, 11, 12, 13, 14]. Pediatric cases include vincristine‐related eruptions, intensive chemotherapy regimens, and postburn skin changes, underscoring that the reaction is not confined to adults [15, 16, 17, 18].

Targeted therapies and several non‐oncologic drugs further broaden the spectrum. BRAF inhibitors such as vemurafenib and dabrafenib can trigger ESS, occasionally in radiation recall‐like or keratosis pilaris‐like distributions, and additional reports implicate fluoropyrimidines, pemetrexed, vincristine, NSAIDs, psychotropics, and pegylated liposomal doxorubicin, often with intertriginous or hand–foot syndrome‐like patterns [19, 20, 21, 22, 23, 24, 25, 26, 27, 28, 29]. Nondrug associations include cytomegalovirus infection and inflammatory or ischemic dermatoses such as panniculitis with pyoderma gangrenosum, annular elastolytic granuloma, and postburn skin [3, 15, 30, 31, 32]. While many reports describe onset within 1–3 weeks of exposure, broader TEC cohorts and pediatric series document a wider window of approximately 2–35 days, with possible recurrences on re‐challenge [13, 17, 18, 33].

### 3.2 Clinical Morphology, Histopathology, and Outcomes

Clinically, ESS most often presents as symmetric erythematous patches or plaques in flexural sites (axillae, inguinal, and cervical folds) or acral areas, within the broader spectrum of TEC and hand–foot syndrome [11, 13, 18, 33]. Lesions may show mild edema, burning, or pain and can mimic intertrigo, cellulitis, acute GVHD, or viral eruptions; papulovesicular and HSV‐like patterns have also been reported [14, 31]. Histologically, the defining feature is duct‐centric squamous metaplasia of eccrine structures, with multilayered squamoid epithelium, intercellular bridges, and variable cornification or apoptosis; secretory coils are often relatively preserved or only subtly involved [1, 2, 5]. Across the literature, most ESS eruptions are self‐limited and respond to supportive care with emollients, cooling, and topical corticosteroids, with or without temporary adjustment of the presumed trigger [2, 13, 33, 34]. Recognizing ESS as a reaction pattern within TEC allows oncologic regimens to be continued or promptly resumed in many cases, while avoiding unnecessary escalation of immunosuppression for misdiagnosed GVHD. Table 1 summarizes indexed ESS cases, and Table 2 provides a clinicopathologic differential diagnosis highlighting key mimickers (Tables 1 and 2).

**TABLE 2:** Clinical and histopathologic differential diagnosis of eccrine squamous syringometaplasia (ESS).

Entity	Typical clinical context and distribution	Key clinical features	Key histopathologic features	Distinguishing points vs. ESS
Eccrine squamous syringometaplasia (ESS)	Oncologic chemotherapy or HSCT conditioning; targeted therapy; less commonly infections (CMV), burns, inflammatory panniculitis; usually intertriginous or acral sites	Symmetric erythematous patches/plaques in folds or acral areas; sometimes hand–foot syndrome–like or TEC‐like; often asymptomatic or mildly painful/pruritic	Duct‐centric squamous metaplasia of eccrine structures, with multilayered squamoid epithelium, intercellular bridges and variable cornification/apoptosis; usually mild perieccrine inflammation; secretory coils relatively preserved or only subtly involved	Reaction pattern centered on eccrine ducts; absence of prominent coil necrosis (NEH) and absence of diffuse interface dermatitis with basal vacuolar change and satellitosis (acute GVHD); architecture benign, without invasive atypia (SCC)
Toxic erythema of chemotherapy (TEC) without prominent ESS	Cytotoxic chemotherapy (e.g., PLD, capecitabine, cytarabine, anthracyclines); HSCT conditioning; folds, palms/soles, pressure areas	Painful erythema and edema of acral sites, folds and contact areas; HFS‐like or intertrigo‐like; may blister or desquamate	Variable vacuolar change, keratinocyte apoptosis and superficial perivascular lymphocytic infiltrate; eccrine apparatus may show injury but without well‐formed squamous metaplasia of ducts	Clinical pattern may overlap ESS, but eccrine changes are less specifically squamous‐metaplastic; eccrine injury can be subtle or absent; histology more epidermotropic and vascular than purely duct‐centric
Neutrophilic eccrine hidradenitis (NEH)	Cytotoxic chemotherapy (classically cytarabine, anthracyclines); leukemia/HSCT; trunk and proximal extremities	Painful erythematous papules/plaques, sometimes fever and systemic symptoms; may mimic cellulitis or TEC	Dense neutrophilic infiltrate targeting eccrine coils with epithelial degeneration and necrosis; neutrophils within eccrine epithelium and lumen; ductal squamous metaplasia usually absent or minimal	Coil‐centric injury with prominent neutrophils and necrosis, not a clean squamous metaplasia; more destructive than ESS and often clinically more tender/systemic
Acute cutaneous GVHD	Allogeneic HSCT, typically within first 100 days; trunk, extremities, sometimes palmar/plantar surfaces	Morbilliform or erythematous maculopapular rash, often starting on trunk; may evolve to erythroderma or blistering; frequently accompanied by gastrointestinal and hepatic involvement	Basal vacuolar alteration with scattered/apoptotic keratinocytes and satellitosis in epidermis and adnexal epithelium; interface dermatitis often extends beyond eccrine units; may show follicular epithelial damage and pigment incontinence	Interface pattern with keratinocyte apoptosis and satellitosis is the key feature; eccrine duct squamous metaplasia is not a defining hallmark. Presence of widespread interface change and adnexal apoptotic bodies favors acute GVHD over ESS
CMV‐associated adnexotropic eruptions	Solid organ or HSCT recipients with CMV viremia; trunk and extremities	Papular or papulovesicular eruption; may coexist with systemic CMV disease (fever, cytopenias, organ involvement)	Eccrine and follicular epithelial involvement with viral cytopathic changes (enlarged cells, nuclear inclusions), mixed inflammatory infiltrate; ESS‐like metaplasia may be present but accompanied by viropathic changes	Viral cytopathic effect and CMV immunohistochemistry/PCR support CMV; ESS‐like changes, if present, are secondary. Clinical context of active CMV infection is crucial
Squamous cell carcinoma/pseudoepitheliomatous hyperplasia (SCC simulators)	Localized plaques/nodules, often on sun‐damaged or scarred skin; no strict temporal link with chemotherapy or HSCT	Hyperkeratotic or nodular lesion; may ulcerate; localized rather than symmetric folds/acral pattern	Irregular, jagged squamous proliferations with cytologic atypia, disordered maturation and possible stromal invasion (SCC) or verruciform pseudoepitheliomatous hyperplasia; adnexal ducts may be engulfed but are not the primary site of metaplasia	Architecture and cytologic atypia differentiate SCC from ESS; ESS remains an adnexal, duct‐centric reactive change with bland cytology and no destructive invasion

Abbreviations: CMV, cytomegalovirus; ESS, eccrine squamous syringometaplasia; GVHD, graft‐versus‐host disease; HFS, hand–foot syndrome; HSCT, hematopoietic stem cell transplantation; NEH, neutrophilic eccrine hidradenitis; PLD, pegylated liposomal doxorubicin; SCC, squamous cell carcinoma; TEC, toxic erythema of chemotherapy.

## 4 Discussion

ESS represents a metaplastic reprogramming of eccrine duct epithelium in response to cytotoxic, ischemic, or inflammatory stress. Concentration of drugs in sweat, high eccrine density in flexural and acral skin, and local occlusion favor duct‐centric injury and squamous transdifferentiation within the spectrum of toxic erythema of chemotherapy [2, 5, 13]. In contrast, acute cutaneous GVHD is characterized by basal vacuolar alteration with scattered apoptotic keratinocytes and satellitosis involving epidermal and adnexal epithelium, often with interface dermatitis extending beyond the eccrine apparatus [34]. In our patient, the epidermis was mildly spongiotic without interface change, and the dominant finding was squamous metaplasia of eccrine ducts with only subtle secretory coil involvement and a sparse perieccrine lymphohistiocytic infiltrate, a profile typical of ESS rather than GVHD. Table 2 summarizes the main clinical and histologic features that help separate ESS from NEH, TEC without prominent ductal metaplasia, viral eruptions, and SCC simulators.

ESS is typically reported during or shortly after chemotherapy/conditioning, but a broader onset window has been documented. In our case, the eruption at ~6 weeks post‐HSCT argues against an isolated immediate conditioning toxicity and supports multifactorial triggers. Eculizumab has not been reported in association with ESS; in fact, no indexed case in our review implicated complement inhibitors as triggers, and mechanistic plausibility is limited. By contrast, CMV has been associated with ESS, and CMV DNA was detectable until eruption [30, 31]. Endothelial injury and capillary leak from transplant‐associated thrombotic microangiopathy may have further lowered the threshold for eccrine metaplasia. Overall, conditioning priming plus CMV and vascular/inflammatory stress likely converged to produce this ESS/SMSE pattern.

Clinicopathologic correlation and duct/coil‐focused assessment are essential to distinguish ESS from NEH, interface‐dominant GVHD, and other mimickers [35, 36]. In pediatric HSCT recipients, biopsy confirmation can avert unnecessary escalation of immunosuppression and support conservative management when appropriate [13, 17].

## 5 Conclusion

ESS is a distinct, duct‐centric metaplasia that can perfectly mimic acute GVHD in HSCT recipients. Histology is decisive and should prompt conservative, supportive care whenever feasible. Our pediatric case reinforces the importance of biopsy‐driven management and adds to the spectrum of ESS mimickers of GVHD in the transplant setting.

## Author Contributions

Conception and clinical data: Benedetta Galli, Cecilia Catapano, and Valeria Boccaletti. Histopathology: Iacopo Ghini. Literature search: Benedetta Galli. Editing and submission: Benedetta Galli. All authors drafted/revised the manuscript and approved the final version.

## Funding

The authors have nothing to report.

## Ethics Statement

The authors have nothing to report.

## Consent

Obtained from legal guardians.

## Conflicts of Interest

The authors declare no conflicts of interest.

## Data Availability

The data that support the findings of this study are available on request from the corresponding author. The data are not publicly available due to privacy or ethical restrictions.
